# Supplementary material for: ProteinShader: illustrative rendering of macromolecules
Source: BMC Struct Biol. 2009 Mar 30;9:19. doi: 10.1186/1472-6807-9-19 (PMC2672931; doi:10.1186/1472-6807-9-19)
Supplement: Additional file 1 — ProteinShader program without source code. This compressed file contains the complete ProteinShader program including associated libraries, but no source code. A README.txt file gives an overview of the ProteinShader distribution, and the index.html file in the help subdirectory has directions on getting started with the program as well as a set of tutorials. [file 1472-6807-9-19-S1.zip › ProteinShader-beta-0_9_4-binary/help/api/org/proteinshader/structure/class-use/Model.html]

Uses of Class org.proteinshader.structure.Model (ProteinShader API)


|  |  |  |
| --- | --- | --- |
| **Overview** \| **Package** \| **Class** \| **Use** \| **Tree** \| **Deprecated** \| **Index** \| **Help** | |  |
| PREV   NEXT | **FRAMES**    **NO FRAMES**     **All Classes** |  |


---


## **Uses of Class org.proteinshader.structure.Model**

| Packages that use Model | |
| --- | --- |
| **org.proteinshader.graphics.adapter** | Holds the StructureToGraphics class, which is used to manage the use of the drawing classes and OpenGL display lists. |
| **org.proteinshader.graphics.displaylists** | Holds the classes needed to manage OpenGL display lists, which are used to cache reusable geometry for spheres, cylinders, ribbon segments, and tube segments. |
| **org.proteinshader.gui** | Holds all of the Swing GUI components and their associated listeners, including class Renderer, which is registered as a listener for the GLCanvas object that is used a drawing surface. |
| **org.proteinshader.gui.components.controlpanel** | Holds the JPanels used in the right-side retractable control panel of the GUI. |
| **org.proteinshader.structure** | Holds the classes that store information from a Protein Data Bank file: Structure, Model, Chain, AminoAcid, Heterogen, Water, Atom, Bond, Helix, BetaStrand, Loop, *etc*. |
| **org.proteinshader.structure.visitor** | Holds Visitor classes that know how to traverse the hierarchy of objects held by class Structure. |

Uses of Model in org.proteinshader.graphics.adapter

| Methods in org.proteinshader.graphics.adapter with parameters of type Model | |
| --- | --- |
| `void` | `StructureToGraphics.cacheSegmentGeometry(GL gl, Model model, StyleEnum style)`             Calls on the SegmentReferences object to cache OpenGL display lists for the Segments of the current Model. |

Uses of Model in org.proteinshader.graphics.displaylists

| Methods in org.proteinshader.graphics.displaylists with parameters of type Model | |
| --- | --- |
| `void` | `SegmentReferences.cacheSegmentGeometry(GL gl, Model model, StyleEnum style)`             Caches an OpenGL display list for each Segment of each Region object in the Model. |

Uses of Model in org.proteinshader.gui

| Methods in org.proteinshader.gui that return Model | |
| --- | --- |
| `Model` | `MediatorImpl.getCurrentModel()`             Returns the current Model. |
| `Model` | `Mediator.getCurrentModel()`             Returns the current Model. |

| Methods in org.proteinshader.gui with parameters of type Model | |
| --- | --- |
| `void` | `MediatorImpl.setCurrentModel(Model model)`             Sets the current Model. |
| `void` | `Mediator.setCurrentModel(Model model)`             Sets the current Model. |
| `void` | `Renderer.setVisibleDrawables(Model model, StyleEnum style)`             An array of opaque Segments, and array of opaque Atoms and Bonds, and an array of translucent Drawable objects will be obtained from the Model. |

Uses of Model in org.proteinshader.gui.components.controlpanel

| Methods in org.proteinshader.gui.components.controlpanel that return Model | |
| --- | --- |
| `Model` | `SelectorPanel.getSelectedModel()`             Returns the currently selected Model from the Model menu. |

| Methods in org.proteinshader.gui.components.controlpanel with parameters of type Model | |
| --- | --- |
| `void` | `SelectorPanel.setChainMenu(Model model)`             Adds the Model's Chains to the Chain menu. |

Uses of Model in org.proteinshader.structure

| Methods in org.proteinshader.structure that return Model | |
| --- | --- |
| `Model` | `Structure.addNewModel(String modelID)`             Creates a Model, adds it to the Structure, and returns a reference to the new Model. |
| `Model` | `Structure.getModel(String modelID)`             Returns the Model with the modelID given as an argument. |

| Methods in org.proteinshader.structure that return types with arguments of type Model | |
| --- | --- |
| `Iterator<Model>` | `Structure.iteratorModels()`             Returns an Iterator for the Models held by this Structure. |

Uses of Model in org.proteinshader.structure.visitor

| Methods in org.proteinshader.structure.visitor with parameters of type Model | |
| --- | --- |
| `void` | `Visitor.visit(Model model)`             Traverses all Chains owned by the Model. |
| `void` | `SFWriterVisitor.visit(Model model)`             Writes all information held by a Model. |
| `void` | `BoundsVisitor.visit(Model model)`             Finds the min and max values for x, y, and z of the Model and then calls setter methods on the Model so that these values are remembered. |
| `void` | `LoopGeneratorVisitor.visit(Model model)`             Generates all Loop objects needed by the Model. |
| `void` | `BondGeneratorVisitor.visit(Model model)`             Generates all standard Bonds (AminoAcids and Waters) for the Model. |

---


|  |  |  |
| --- | --- | --- |
| **Overview** \| **Package** \| **Class** \| **Use** \| **Tree** \| **Deprecated** \| **Index** \| **Help** | |  |
| PREV   NEXT | **FRAMES**    **NO FRAMES**     **All Classes** |  |


---

# *Copyright © 2007-2008*
